# Subclinical alexithymia modulates early audio-visual perceptive and attentional event-related potentials

**DOI:** 10.3389/fnhum.2014.00106

**Published:** 2014-03-03

**Authors:** Dyna Delle-Vigne, Charles Kornreich, Paul Verbanck, Salvatore Campanella

**Affiliations:** Laboratoire de Psychologie Médicale et d'Addictologie, ULB Neuroscience Institute, CHU Brugmann-Université Libre de BruxellesBrussels, Belgium

**Keywords:** event-related potentials (ERPs), bimodal, alexithymia, emotion, subclinical

## Abstract

**Introduction:** Previous studies have highlighted the advantage of using audio–visual oddball tasks (instead of unimodal ones) in order to electrophysiologically index subclinical behavioral differences. Since alexithymia is highly prevalent in the general population, we investigated whether the use of various bimodal tasks could elicit emotional effects in low- vs. high-alexithymic scorers.

**Methods:** Fifty students (33 females and 17 males) were split into groups based on low and high scores on the Toronto Alexithymia Scale (TAS-20). During event-related potential (ERP) recordings, they were exposed to three kinds of audio–visual oddball tasks: neutral-AVN—(geometrical forms and bips), animal-AVA—(dog and cock with their respective shouts), or emotional-AVE—(faces and voices) stimuli. In each condition, participants were asked to quickly detect deviant events occurring amongst a train of repeated and frequent matching stimuli (e.g., push a button when a sad face–voice pair appeared amongst a train of neutral face–voice pairs). P100, N100, and P300 components were analyzed: P100 refers to visual perceptive and attentional processing, N100 to auditory ones, and the P300 relates to response-related stages, involving memory processes.

**Results:** High-alexithymic scorers presented a particular pattern of results when processing the emotional stimulations, reflected in early ERP components by increased P100 and N100 amplitudes in the emotional oddball tasks [P100: *F*_(2, 48)_ = 20,319, *p* < 0.001; N100: *F*_(2, 96)_ = 8,807, *p* = 0.001] as compared to the animal or neutral ones. Indeed, regarding the P100, subjects exhibited a higher amplitude in the AVE condition (8.717 μV), which was significantly different from that observed during the AVN condition (4.382 μV, *p* < 0.001). For the N100, the highest amplitude was found in the AVE condition (−4.035 μV) and the lowest was observed in the AVN condition (−2.687 μV, *p* = 0.003). However, no effect was found on the later P300 component.

**Conclusions:** Our findings suggest that high-alexithymic scorers require heightened early attentional resources in comparison to low scorers, particularly when confronted with emotional bimodal stimuli.

## Introduction

Event-related potentials (ERPs) offer a sensitive method of monitoring brain electrical activity, through a high temporal resolution in the order of milliseconds (Rugg and Coles, 1995). By this way, it is possible to observe the different electrophysiological components needed for healthy subjects to reach a “normal” performance during distinct cognitive tasks. ERPs also allow identifying the electrophysiological component(s) associated with the onset of a dysfunction in pathological populations. In this regard, ERPs constitute an unique technique for investigating even minor cognitive limitations, which might be undetectable at the behavioral level (e.g., Maurage et al., 2009), as indexing the neuro-cognitive origin of a deficit may give to clinicians important indications about the relevant impaired cognitive stages that should be rehabilitated (Campanella, 2013).

Among others, a classical paradigm used to evoke ERP components consists in an “oddball target detection task,” where the subject has to detect as quickly as possible (typically by pressing a button) a deviant-rare stimulus (e.g., a 1000 Hz sound) among a train of frequents stimuli (e.g., a 500 Hz sound). The oddball task evokes robust and reliable phenomena that have been used as markers of cognitive function (Rodríguez Holguín et al., 1999). There is increasing evidence to support that a number of early and late neuroelectric features in the information-processing stream can be anomalous in various psychiatric populations, the common finding being P300 abnormalities (Hansenne, 2000). The P3b component arises approximately 300–350 ms after an auditory stimulus and from 400 to 450 ms following visual input in the centro–parietal areas of subjects detecting deviant stimuli in oddball tasks (i.e., when the subject has to actively evaluate, categorize, and make decisions regarding relevant stimuli). The discrimination of the target from the standard stimulus (deviant minus frequent) generates a robust P300, due to the novelty of the deviant stimulus, compared to the repeated frequent ones. (Polich, 2007). This positive deflection appears to be related to memory processing (Polich, 2007), since its amplitude does not seem to be modulated either by the motor requirements of the task or the physical features of the stimulus (Duncan et al., 2009). However, although a large number of studies have provided evidence on the relevance of the P300 component as a biological marker of mental illness, its clinical sensitivity has been hampered by the fact that its parameters (amplitude and latency) are diagnostically unspecific and unreliable (Pogarell et al., 2007). In other words, even though differences in P300 amplitude and latency can indicate the severity or evolution of a clinical state, the clinical value of this component as a diagnostic index is low. Therefore, a current and important challenge for neurophysiologists is to discover novel and appropriate procedures for enhancing the clinical applicability and sensitivity of the P300 component (e.g., Bruder et al., 2009).

For this purpose, we recently proposed the use of more ecological stimulations (namely synchronized congruent audio-visual stimuli) in oddball tasks, instead of unimodal stimulations. In these studies (2010; 2012), two groups of participants were compared: one group was composed entirely of healthy individuals, whereas the other consisted of healthy people displaying anxious and depressive tendencies in the absence of full-blown clinical symptoms. Both groups were submitted to unimodal (visual and auditory separately) and bimodal oddball tasks. The principal findings suggested that although the two study groups differed in their subclinical level of comorbid anxiety and depression, this difference could not be detected by their P300 amplitudes during unimodal visual and auditory oddball tasks; however, *bimodal tasks did allow for the detection of subclinical symptoms*. The investigators hypothesized that the bimodal processing of multimodal information involves complex associative processes, including the integration of unimodal visual and auditory products into a single, coherent representation (Campanella and Belin, 2007 for a review). Therefore, a deficit in these associative processes might explain why the bimodal procedure enabled detection of a subclinical difference, which was impossible during unimodal conditions.

Nevertheless, despite the potential relevance of these studies to be applied in real clinical settings, the findings raised several questions, two of which formed the background for the present study. First, in the study by Campanella et al. (2012), individuals with subclinical anxious-depressive tendencies were compared to healthy subjects using two kinds of bimodal oddball tasks, consisting of emotional and neutral conditions (i.e., geometric forms and simple sounds) and two unimodal tasks (visual and auditory). In contrast to the unimodal tasks, both bimodal conditions elicited P300 amplitude differences (i.e., lower P300 amplitudes in the subclinical group compared to the control group), which were independent of the emotional content of the stimulations. In other words, the bimodal P300 helps to disclose differences between subclinical and control participants that did not appear through unimodal tasks; however, no major difference emerged from the emotional-bimodal P300, as compared with the non-emotional one. This was quite surprising, as many psychiatric diseases show a deficit in emotional processing, which is indexed by decreased performance rates, longer response latencies compared to controls (Power and Dalgleish, 1997), as well as by decreased and delayed P300 component (Campanella et al., 2002, 2005, 2006; Mejias et al., 2005; Rossignol et al., 2005, 2008, 2012; Maurage et al., 2007a, 2008). Therefore, we would have expected to have greater bimodal P300 differences between groups when emotional stimuli were used. A possible explanation for this absence of effect could be that a major affective dimension, well-known to affect emotion processing, was not taken into account in these previous studies, i.e., alexithymia. In fact, alexithymia is often considered to be a stable personality trait that reflects a deficit in the conscious experience of emotion (Lane et al., 2000), involving an inability to recognize, regulate, and describe emotions (Luminet et al., 2001; Swart et al., 2009). Alexithymia can also describe difficulties in differentiating feelings from bodily sensations and decreased capacity for symbolization of emotion, such as in fantasy or dreams (Sifneos, 1973; Bagby and Taylor, 1997). Currently, this disorder is considered to be a risk and/or maintenance factor for various medical and psychiatric conditions, including anxiety and depression, even at a subclinical level (e.g., Berthoz et al., 1999; Luminet et al., 2001). Therefore, some differences between the non-emotional and the emotional bimodal oddball tasks might have been obscured in our previous study (Campanella et al., 2012) since we did not measure this factor. In the present study, we added the measure of alexithymia to verify this hypothesis.

Second, it is currently well accepted that ERP data should not “exclusively” focus on the P300 component, as numerous studies have shown that P300 deficits may be correlated with previous “early” ERP alterations (Maurage and Campanella, 2013). For instance, Maurage et al. (2007a) demonstrated in a visual–emotional oddball task that P300 modulations were correlated with deficits in earlier P100 and N170 components. Similarly, a previous study by our group (Campanella et al., 2006) confirmed earlier results obtained by Foxe et al. (2001), which indicated that schizophrenic patients displayed reduced amplitude and longer latencies in early visual components (e.g., P100, N100, and N170). The necessity to investigate other ERP components than the P300 was also supported by the fact that, up to now, reports describing the influence of alexithymia on the P300 component have been inconsistent. While some authors have described no effect of alexithymia on P300 amplitudes (Vermeulen et al., 2008; Walker et al., 2011), others have reported distinct P300 alterations in alexithymic individuals. For example, Bermond et al. (2008) found larger P300 amplitudes related to emotional stimuli in comparison to neutral stimuli, as well as for women compared to men. Also, they elicited a significant gender x alexithymia effect: high-scoring females on TAS-20 exhibited reduced P300 amplitudes compared to low-alexithymic women for negative pictures, whereas the opposite was true for males. In addition, Pollatos and Gramann (2011) reported lower P300 amplitudes for high-scorers on the TAS-20 in response to unpleasant pictures, while Franz et al. (2004) obtained a contradictory result.

In the present study, we intend therefore to analyze bimodal P300 components as well as earlier bimodal P100 and N100 components. Both N100 and P100 components are related to visual and auditory stimuli, but the N100 is more related to the auditory processing (e.g., Jessen et al., 2012; Liu et al., 2012), while the P100 is more visual (e.g., Allison et al., 1999; Singhal et al., 2012; Peschard et al., 2013). Specifically, the P100 wave is a positive component recorded around 100 ms at occipital sites in response to any visual stimulation and is associated with early spatial attention processes. It reflects activity in the extra-striate visual cortex and is sensitive to top–down control mechanisms (Martínez et al., 1999). As a result, the P100 wave is related to the physical–visual properties of stimuli, constituting a more automatic perceptual level of the information-processing stream (Heinze and Mangun, 1995). Usually, when emotional images are displayed, the early/sustained attention increases, easing the effect of the stimuli. This outcome is echoed by amplitude modulations in ERPs, especially the P100 component (Singhal et al., 2012). Regarding the N100 component, this negative deflection peaks around 100 ms following the onset of a prosodic stimulus. It is generated in the bilateral secondary auditory cortex (Engelien et al., 2000) and reflects the extraction of acoustic cues (frequency and intensity) during early auditory treatment. Typically, its amplitude increases based on the amount of attention allocated to an acoustic stimulus (Alho et al., 1994; Rinne et al., 2005).

Some electrophysiological data linking alexithymia to early ERPs abnormalities already existed, but involved, to our knowledge, only visual or auditory stimulations separately. Pollatos and Gramann (2011) reported that processing emotional pictures led to reductions in P100 amplitudes for high-scorers on the TAS-20. This finding was especially associated with neutral and positive pictures, with early P100 deficits linked to later variations in P300 amplitudes. Also, when subjects with subclinical tendencies of alexithymia were asked to detect and identify deviant stimuli in emotional prosody, Goerlich et al. (2012) found N100 alterations in high TAS-20 scorers. In particular, larger N100 amplitudes were observed in relation to disgusted prosody, whereas no behavioral differences were found between the two groups (also see Schäfer et al., 2007). Overall, it appears that alexithymic subjects may present a global deficit in perceiving all kinds of basic emotions, and these impairments can be neurophysiologically indexed by disrupted visual (P100 amplitude) and/or auditory (N100 amplitude) processing. These deficiencies likely extend not only to static and dynamic emotional facial expressions (EFEs) (Kätsyri et al., 2008), but also to neutral facial expressions (Montebarocci et al., 2011). Likewise, alexithymic subjects display a diminished capacity to recognize emotions related to non-verbal stimuli and responses (Lane et al., 1996). Since spatially-degraded or briefly-presented (≤1 s) stimuli are more difficult to interpret for alexithymic subjects, it has been suggested that these individuals may need more time and/or more information to correctly identify EFEs (Franz et al., 2004, 2008; Parker et al., 2005; Kätsyri et al., 2008; Prkachin et al., 2009).

Overall, the main objective of the present study was to determine: (1) whether subclinical alexithymia results in particular emotional effects (i.e., amplitude and/or latency modulations) during the performance of emotional bimodal oddball tasks compared to non-emotional ones; and (2) whether modulations on the P300 amplitude and/or on earlier components can be observed under these conditions. In order to reach this double objective, participants will be confronted to bimodal emotional and non-emotional (geometric forms and simple sounds) oddball tasks, similar than those used in Campanella et al.'s study (2012). However, we also added an “animal” bimodal condition, as this feature allowed us to have an authentic bimodal semantic association, which involved “non-emotional” stimuli existing in the subject's environment (i.e., a dog barking and a cock crowing). Indeed, this “meaningful” but “non-emotional” condition, is considered as a semantic condition, which is known to produce higher neural responses (Bookheimer, 2002). We postulated that emotional stimulation would require more processing than other types of stimuli, irrespective of the alexithymia, as emotional conditions command attentional resources and are prioritized due to significance (Vuilleumier, 2005). Therefore, when analyzing the effect of alexithymia, we believe that an “emotional effect” might exist, meaning that high scorers on the TAS-20 would experience more difficulties in processing emotional conditions compared to low scorers. However, no differences would be expected between the emotional and animal stimuli for the low-scoring group. Also, we hypothesize that deficiencies in the alexithymia-high group should be associated with higher early ERP components (P100 and/or N100 amplitudes), as more attentional resources will be required to accomplish tasks (e.g., Franz et al., 2004). We have to outline that we only used negative (sad) and neutral stimuli in the emotional condition because Mann et al. (1994) reported that high-scorers performed especially worse than low-scorers in labeling tasks when sad pictures were presented. Also, alexithymia is negatively correlated with the propensity to experiment positive emotions (Bagby et al., 1994b), and some neuroimaging studies tend to confirm this fact. For example, Zald (2003) described improved cerebral features when using negative stimulations. Mantani et al. (2005) reported reduced activation of the posterior cingulate cortex in response to past and future happy situations in high alexithymic subjects, and Eichmann et al. (2008) found that masked sad faces were associated with greater bilateral activation of the fusiform gyrus in this kind of subjects. Furthermore, Zhang et al. (2012) highlighted that alexithymic subjects were particularly affected at the neural level when identifying negative emotions (anger, sadness, and fear). We also propose that the neutral conditions, having no “semantic content,” will be the easiest to process for both groups. Finally, we found it interesting that some studies failed to exhibit performance differences between healthy and clinical/subclinical alexithymic populations. For example, in a study by Mann et al. (1995), high-alexithymic substance abusers performed similarly to a control group in labeling EFEs. Pandey and Mandal (1997) failed to elicit behavioral differences between high and low TAS-20 scorers using a labeling task with sadness, happiness, fear, anger, disgust, and surprise EFEs. Also, Galderisi et al. (2008) did not find differences between patients with panic disorder and high TAS-20 scores when compared to healthy controls. Therefore, as no behavioral difference was expected between groups (mainly due to the facility of the oddball tasks), we hypothesized that any alexithymia-related effect will be disclosed by modulations on the response-related stages, indexed by P300 component, as in Vermeulen et al. (2008).

## Methods

### Participants

Fifty students (33 females and 17 males) enrolled at the Free University of Brussels (18–27 years old) participated in this study. These individuals displayed normal/corrected vision and normal hearing. In addition, they were not on any medications and had no history of neurological/psychiatric disease. Screened through questionnaires, students presenting a heavy social drinking behavior, using drugs (mainly cannabis), or smoking >10 cigarettes per day were excluded from the study, as these variables are known to affect the P300 component (Solowij et al., 1995; Polich and Criado, 2006; Mobascher et al., 2010). The local ethics committee at the Brugmann Hospital approved the study, and informed written consent was obtained from each participant.

### Task and procedure

Before the ERP task, the subjects were asked to complete various self-reported questionnaires: the Beck Depression Inventory (13 items, BDI, Beck and Steer, 1987; French version: Collet and Cottraux, 1986) to assess depression tendencies; scores between 0 and 4 signify absence of depression, while scores between 8 and 15 displayed a subclinical level of moderate depression (Beck and Beck, 1972); the State and Trait Anxiety Inventory (STAI, Spielberger et al., 1983; French version: Bruchon-Schweitzer and Paulhan, 1993) for anxiety tendencies; scores below 36 reflect a very low anxiety, 36–45 low anxiety, 46–55 a normal anxiety, 56–65 high, and more than 65 very high anxiety; and the TAS-20 (Bagby et al., 1994a,b; French version: Loas et al., 1996) to measure alexithymic propensities; a score of less than 51 is not considered as alexithymia, and a score equal or higher than 61 indicates alexithymia (Taylor et al., 1997). The TAS-20 contains 20 items, each rated on a 5-point Likert scale. The test is composed of 3 subscales, focusing on difficulties in identifying feelings, difficulties in describing feelings, and an assessment of externally-oriented thinking style.

**Figure 1:**
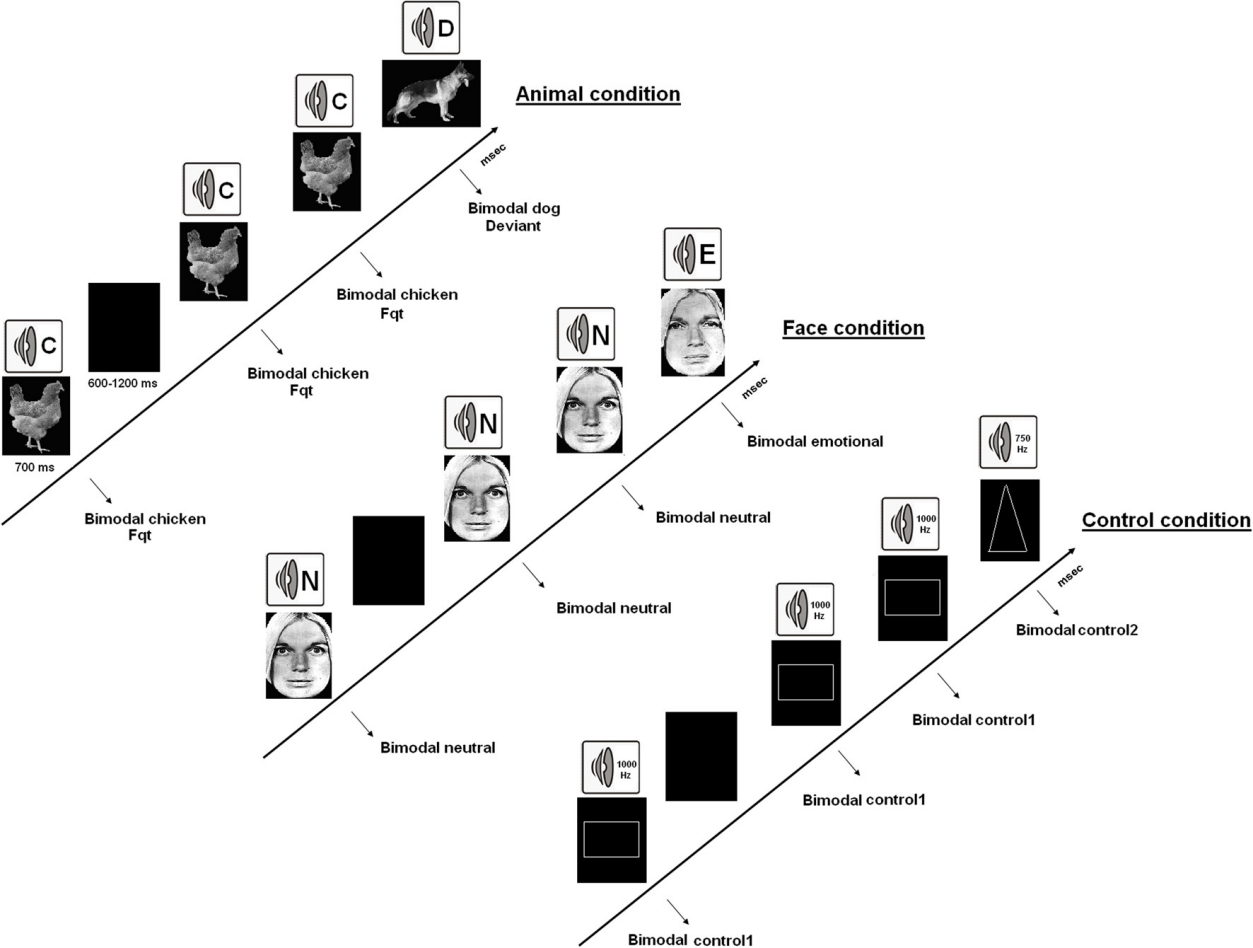
**The 3 kinds of bimodal oddball tasks**. AVA, which combined synchronized presentations of animal pictures and sounds; AVE, which combined faces and voices displaying neutrality or sadness; and the AVN control condition, which used simple geometrical figures and sounds.

All participants carried out 6 bimodal (synchronized presentations of visual and auditory stimulations) oddball tasks [two “emotional” (AVE), two “animal” (AVA), and two “neutral” (AVN) tasks]. In each exercise, the participants were required to detect as quickly as possible deviant events occurring amongst a train of repeated and frequent matching stimuli by clicking a button with their right forefinger. This experimental set-up is similar to that used in Campanella et al. (2010), (2012). In the “emotional” bimodal auditory–visual oddball task (AVE), pairs of synchronized and congruent faces and voices were displayed to participants [frequent stimulus: a neutral face and a neutral voice pronouncing the word “*papier*” (French for “paper”); deviant stimulus: a sad face with a sad voice; the frequent and deviant stimuli were inverted in the second block]. Faces were selected from Ekman and Friesen's set of standardized pictures (1976), and voices were chosen from the validated battery of vocal emotional expressions (Maurage et al., 2007b). In the “animal” bimodal auditory–visual oddball task (AVA), pairs of synchronized and congruent pictures of a dog and a cock with their respective shouts were displayed to participants (again, in the two blocks of this condition, the frequent stimulus and the deviant one were inverted). Finally, in the “neutral” bimodal auditory–visual oddball task (AVN), pairs of synchronized geometrical figures and tones were shown to participants (frequent stimulus: a square and a 750 Hz sound; deviant stimulus: a triangle with a 1000 Hz sound; stimuli were once again inverted in the second block). The various stimuli are illustrated in Figure 1.

Each block included a total of 130 stimuli (100 frequent and 30 deviant), and every participant completed 6 blocks (2 emotional, 2 animal, and 2 neutral; approximately 5 min for each block). Participants were informed about upcoming exercises during the intervals between the blocks. The order of the 6 blocks was counterbalanced among the subjects.

During the ERP recordings, each participant sat alone in a dark room on a chair placed one meter from the screen, with his or her head restrained on a chin rest. The visual stimuli subtended a visual angle of 3° × 4°. Each stimulus was presented for 700 ms, and a black screen was displayed between stimuli for a random duration (600–1200 ms). From the onset of each stimulus, the participants had at least 1300 ms to respond. Reaction times and error rates were recorded. There were two categories of errors: omission (i.e., not pressing the answer key when a deviant stimulus appeared) and false recognition (i.e., pressing the answer key when a standard stimulus appeared). Participants were informed that speed was important, but not at the cost of accuracy. Only correct answers (i.e., deviant stimuli for which the subject pressed the answer key) were used in the analysis of reaction times and ERPs.

### EEG recording and analysis

The electroencephalography (EEG) was recorded by 32 electrodes mounted in an electrode Quick-Cap. Electrode positions included the standard 10–20 system locations and intermediate positions. Recordings were made with a linked mastoid physical reference but were re-referenced using a common average (Bertrand et al., 1985). The EEG was amplified by battery-operated A.N.T.® amplifiers with a gain of 30,000 and a band-pass of 0.01–100 Hz. The impedance of the electrodes was kept below 20 k'ω. The EEG was continuously recorded (sampling rate 500 Hz; A.N.T. Eeprobe software) and trials that were contaminated by electrooculogram (EOG) artifacts (mean of 15%) were eliminated offline, using a procedure developed by Semlitsch et al. (1986). In brief, an average artifact response was computed for each individual based on a percentage of the maximum eye movement potential (generally recorded on prefrontal electrodes). The EOG response was thereby subtracted from the EEG channels on a sweep-by-sweep, point-by-point basis in order to obtain ocular artifact-free data. Epochs beginning 200 ms prior to the stimulus onset and continuing for 800 ms were created. Three parameters were coded for every stimulus: (1) the modality of the task (AVE, AVA, and AVN), (2) the type of stimulus (deviant vs. frequent), and (3) the response type (keypress for deviant stimuli, no keypress for frequent ones). Data were filtered with a 30 Hz, low-pass filter.

**Figure 2:**
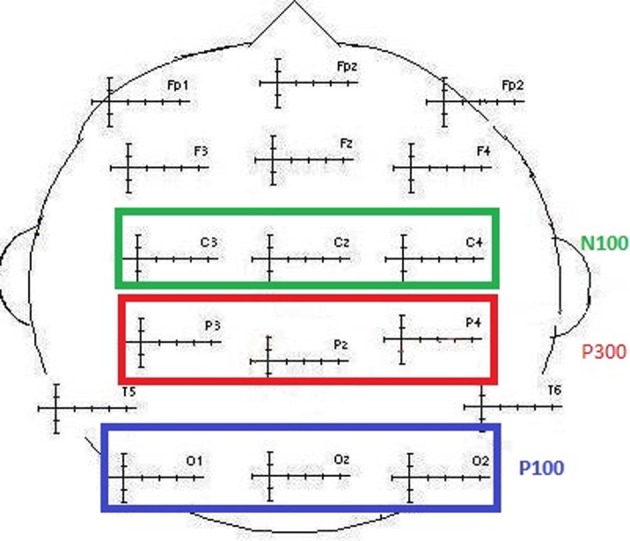
**Illustration of the electrodes classically selected to collect N100, P100, and P300 values (amplitude and latency)**.

For each modality and each subject, the component of interest (P100, N100, and P300) was investigated by gathering individual values of the maximum peak amplitudes and latencies separately for frequent and for deviant stimuli. These amplitudes were further averaged when no effect of the electrodes was found. These data were obtained from the classic electrodes used to define the P100 component, recording the maximum amplitudes (O1, Oz, and O2; maximal peak values between 90 and 160 ms). The same was true for the N100 (C3, Cz, and C4; maximal peak values between 90 and 160 ms) and the P300 (P3, Pz, and P4; maximal peak values between 250 and 600 ms) components (see Figure 2). The data were explored using repeated measures of analysis of variance (ANOVA) with the Greenhouse–Geisser correction applied when appropriate, using S.P.S.S. 21.0®.

## Results

### Behavioral data

The participants' responses were 99.8% correct. Therefore, only the correct response latencies were statistically analyzed. The characteristics of the sample are shown in Table 1.

**Table 1:** **Means and standard deviations (in parentheses) for the whole sample (*n* = 50) for age, BDI and STAI and TAS-20 psychological tests**.

***n* = 50**	**Mean**	***S.D***
Age	21.02	2.095
BDI	5.68	5.464
STAI A	50.22	13.826
STAI B	48.68	12.953
TAS-20	48.74	10.583
F1 tas	18.16	5.64
F2 tas	14.98	4.48
F3 tas	17.18	10.58

To examine whether the TAS-20 scores had an influence on the subjects' performances, we computed an ANCOVA on response times (RTs) for correct responses with modality (neutral, animal, and emotional) set as within-variables and inventory scores (BDI, STAI, and TAS-20) as covariates. We also performed Pearson's correlations between the different tests. It appeared that all of the tests were intercorrelated, except for the TAS-20 (see Table 2). We observed a significant modality effect [*F*_(2, 90)_ = 6.182, *p* = 0.003] but no effect of the covariates (*p* > 0.05). *Post-hoc* Bonferroni tests revealed longer reaction times for the emotional stimuli (449 ms) compared to the animal condition (396 ms), whereas the shortest RTs were observed for neutral stimuli (384 ms). Results are shown in Tables 2 and 3.

**Table 2:** **Correlations between the different covariates (*n* = 50)**.

	**BDI**	**STAI-A**	**STAI-B**	**TAS-20**
BDI				0.234
*p*				0.103
STAI-A	0.695			0.078
*p*	<0.001			0.592
STAI-B	0.788	0.766		0.176
*p*	<0.001	<0.001		0.223
TAS-20	0.234	0.078	0.176	
*p*	0.103	0.592	0.223	

**Table 3:** **Means and standard deviations (in parentheses) of reaction times (ms) for “emotional” bimodal-AVE, “animal” bimodal-AVA and “neutral” bimodal task-AVN, independently of the group**.

**Condition**	**Reaction times (ms)**
AVN	384
	(58.74)
AVA	396
	(53.94)
AVE	449
	(56.99)

**Figure 3:**
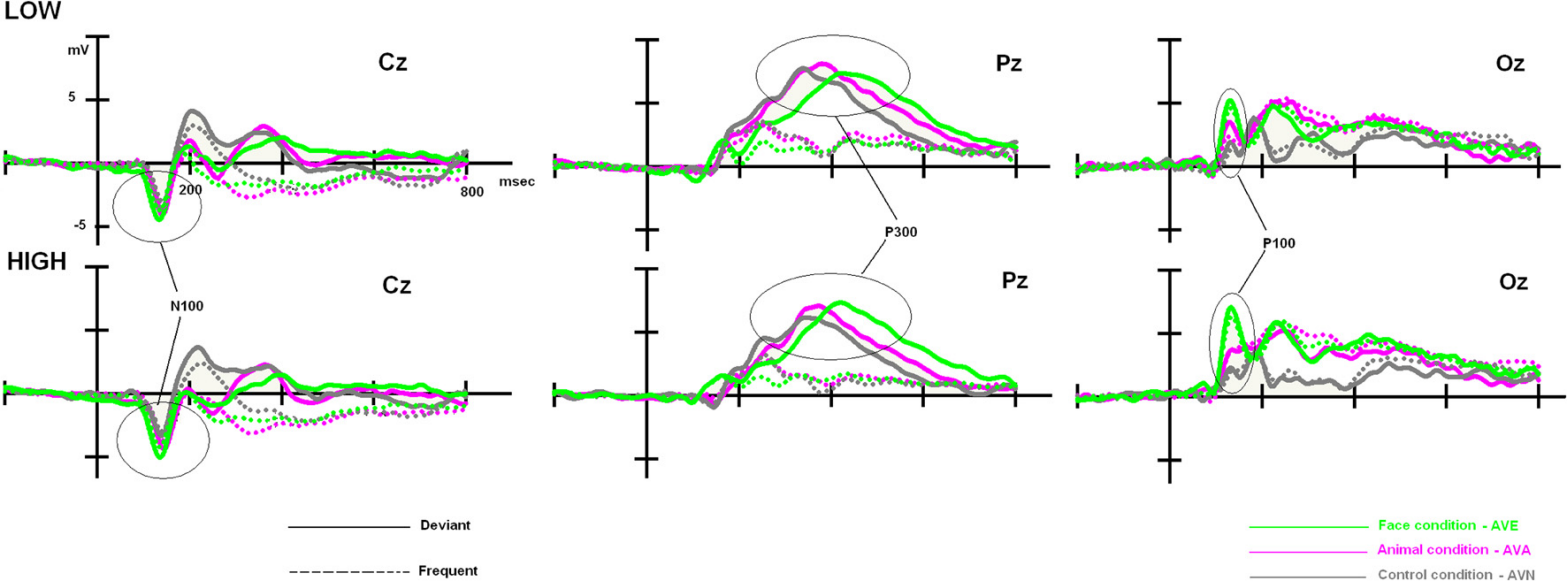
**Low- and high-alexithymic scores related to the three ERPs of interest (P100, N100, and P300), which were recorded during each bimodal task on their respective electrodes**.

Although, as expected, no differences related to subclinical alexithymia were observed with the RTs, it might be interesting to compute statistical analyses based on the amplitude and latency values of the ERP components. Indeed, ERPs have been shown to detect even minor neurocognitive restrictions, which are undetectable at the behavioral level (Maurage et al., 2009; Campanella et al., 2012). These further analyses are presented below, and ERP components of interest (P100, N100, and P300) are illustrated in Figure 3.

### ERP data

#### P100

We computed an ANCOVA on P100 amplitudes, with stimulus (frequent and deviant), electrodes (O1, Oz, and O2), and modality (neutral, animal, and emotional) set as within-variables. Inventory scores (BDI, STAI, and TAS-20) served as covariates. Using this analysis, we found some significant interactions with alexithymia. In particular, we observed a tendency for modality × TAS [*F*_(2, 90)_ = 3.094, *p* = 0.061] and a significant interaction for stimulus × TAS [*F*_(1, 45)_ = 4.538, *p* = 0.039], while the triple interaction for modality × stimulus × TAS was not significant [*F*_(2, 90)_ = 2.37, *p* = NS]. The other covariates (BDI and STAI) did not show significant effects. We also performed Pearson's Correlations between the covariates, according to the groups, and all tests were intercorrelated, except for the TAS-20 (see Table 4).

**Table 4:** **Correlations between the different covariates according to the groups**.

	**BDI**	**STAI-A**	**STAI-B**	**TAS-20**
**LOW**				
BDI		0.624	0.816	0.227
*p*		0.001	<0.001	0.276
STAI-A			0.636	0.310
*p*			0.001	0.131
STAI-B				0.290
*P*				0.160
TAS-20	0.2274	0.310	0.290	
*P*	0.276	0.131	0.160	
**HIGH**				
BDI		0.761	0.768	0.074
*p*		<0.001	<0.001	0.726
STAI-A			0.891	−0.073
*p*			<0.001	0.727
STAI-B				−0.041
*p*				0.845
TAS-20	0.074	−0.73	−0.41	
*p*	0.726	0.727	0.845	

To clarify the significant stimulus × TAS interaction, the participants (*n* = 50) were split into LOW and HIGH groups based on their TAS-20 scores, and divided according to the stimulus type (frequent vs. deviant). We performed an ANCOVA on P100 amplitudes, with electrodes (O1, Oz, and O2), and modality (neutral, animal, and emotional) set as within-variables. Inventory scores (BDI, STAI, and TAS-20) served as covariates. For frequents, we obtained a non-significant result [modality × TAS = *F*_(2, 90)_ = 1.446, *p* = NS]. No other covariates (BDI and STAI) effects were found. For deviants, we obtained a significant modality × TAS interaction [*F*_(2, 90)_ = 4.392, *p* = 0.019]. Further analyses were thus computed for deviants only. We divided the sample into two groups, based on the median TAS-20 value: low alexithymic scores (TAS-20 ≤ 50; *n* = 25) and high-scorers (TAS-20 > 50; *n* = 25). These two groups displayed alexithymia differences [*t*_(48)_ = −10.980, *p* < 0.001] but were matched in gender [χ^2^_(1)_ = 0.089, *p* = NS], which is well-known to modulate early visual ERP components during emotion processes (e.g., Proverbio et al., 2006), and age [*t*_(48)_ = 1.152, *p* = NS]. The data are summarized in Table 5. Therefore, we computed a 2 × 3 × 3 ANOVA on P100 amplitude values, with group (LOW and HIGH) set as the between-subject factor and modality (AVE, AVA, and AVN) and electrode (O1, Oz, and O2).

**Table 5:** **Means and standard deviations (in parentheses) of the low group (LOW) and high (HIGH) groups' scores for age, BDI and STAI and TAS-20 psychological tests, and reaction times (ms) for the 3 conditions**.

	**Gender (F/M)**	**Age**	**BDI**	**STAI-A**	**STAI-B**	**TAS-20**	**AVN RT**	**AVA RT**	**AVE RT**
LOW	(17/8)	21.36	4.68	50	47.16	**39.88**	382	394	447
		(2.307)	(4.862)	(12.806)	(13.133)	(6.24)	(58.373)	(54.05)	(55.421)
HIGH	(16/9)	20.68	6.68	50.44	50.2	**57.6**	386	398	451
		(1.842)	(5.935)	(15.039)	(12.855)	(5.115)	(60.24)	(54.862)	(59.599)
*p*	0.765	0.255	0.199	0.912	0.412	**<0.001**	0.999	0.999	0.999

*Significant results are indicated in bold*.

Once again, we observed a significant effect of modality [*F*_(2, 96)_ = 26.630, *p* < 0.001]. *Post-hoc* Bonferroni tests showed that the distinct conditions were associated with differing P100 amplitudes (independent of the group). The highest amplitude was associated with the AVE condition (7.521 μV, *p* < 0.001), whereas the lowest amplitude was observed during the AVN condition (4.279 μV, *p* < 0.001). AVA (5.393 μV) differed from both AVN (*p* = 0.037) and AVE (*p* < 0.001) conditions. We also observed a significant modality × group interaction [*F*_(2, 96)_ = 3.649, *p* = 0.030]. In order to better characterize this interaction, further analyses were conducted on the averaged amplitudes (mean amplitude of O1, Oz, and O2), as no electrode effects were found [modality × electrode: *F*_(4, 192)_ = 1.593, *p* = NS; modality × electrode × group: *F*_(4, 192)_ = 0.745, *p* = NS].

**Figure 4:**
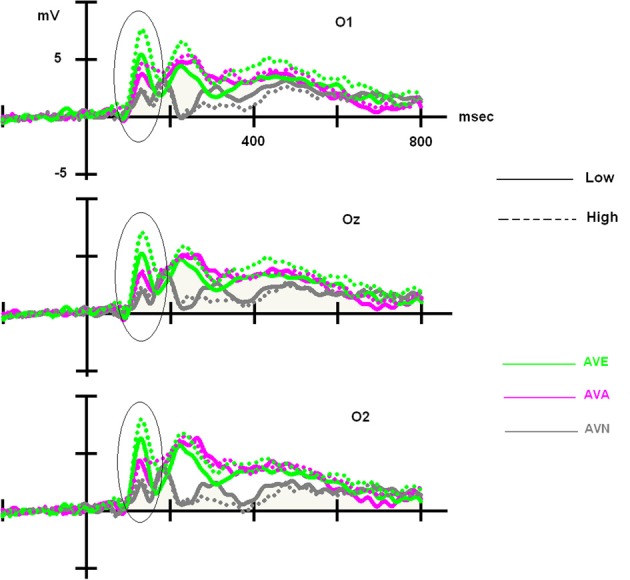
**Main effect observed on the P100 component for deviant stimuli: alexithymia-high scoring individuals displayed enhanced amplitudes for the AVE condition compared to low-scorers, while no difference emerged for the AVA and AVN conditions**. Please note that independently of the group, AVE and AVA stimuli generated higher P100 amplitudes than AVN.

For the LOW group, an ANOVA 3 with modality set as the within-variable revealed a significant effect of modality [*F*_(2, 48)_ = 7.181, *p* = 0.002]. *Post-hoc* Bonferroni tests showed that the highest amplitude was observed in the AVE condition (6.324 μV), which differed significantly from that in the AVN condition (4.177 μV, *p* = 0.004). No differences were found between the AVE and AVA (5.210 μV, *p* > 0.05) conditions or the AVN and AVA conditions (*p* > 0.05). For the HIGH group, the significant effect of modality was also demonstrated [*F*_(2, 48)_ = 20.319, *p* < 0.001]. *Post-hoc* Bonferroni tests showed that the highest amplitude was found in the AVE condition (8.717 μV), which was significantly different from that observed during the AVN condition (4.382 μV, *p* < 0.001). In this group, the AVA amplitude (5.576 μV) was also significantly lower than the AVE amplitude (*p* < 0.001), suggesting a specific emotional effect. However, the AVA condition was not significantly different from the AVN (*p* > 0.05). These P100-related differences between LOW- and HIGH-alexithymic scorers are illustrated in Figure 4 and means and standard deviations are found in Table 6.

**Table 6:** **Means and standard deviations (in parentheses) of P100, N100 and P300 amplitudes for frequent and deviant stimuli, in the “emotional” bimodal-AVE, “animal” bimodal-AVA and “neutral” bimodal task-AVN, for each group**.

**Condition**	**P100 Fqt**	**P100 Dev**	**N100 Fqt**	**N100 Dev**	**P300 Fqt**	**P300 Dev**
**LOW**						
**AVN**	2.815 (2.863)	**4.177 (2.503**)	−2.977 (1.843)	−3.671 (2.352)	2.342 (1.434)	6.552 (2.576)
**AVA**	4.146 (2.917)	5.21 (3.287)	−2.896 (1.635)	−3.300 (1.781)	3.158 (2.008)	7.087 (3.840)
**AVE**	5.648 (3.414)	**6.324 (2.809)**	−3.269 (1.393)	−3.856 (1.464)	2.583 (1.422)	6.401 (2.758)
**HIGH**						
**AVN**	3.798 (2.552)	**4.382 (3.103)**	−**2.482 (1.260)**	−**2.893 (2.137)**	1.549 (1.431)	5.886 (2.717)
**AVA**	5.098 (3.339)	**5.576 (3.974)**	−**3.104 (1.267)**	−**3.824 (1.569)**	2.782 (2.657)	7.157 (2.730)
**AVE**	7.706 (4.971)	**8.718 (5.389)**	−**3.720 (1.811)**	−**4.350 (1.855)**	2.783 (1.844)	6.935 (2.968)

*Significant amplitudes are indicated in bold*.

Taken together, no behavioral differences emerged between the two groups. Moreover, in the LOW group, we observed that the AVA and AVE deviant stimuli were handled identically (no emotional specificity), both producing higher P100 amplitudes than the neutral stimuli (AVN). However, in the HIGH group, amplitudes during the AVE condition were higher than those in the AVA and AVN conditions, suggesting that more attentional resources were required to handle the emotional stimuli. Similar analyses were conducted on P100 latencies, but no significant results were obtained (*p* > 0.05).

### N100 amplitudes

We analyzed the N100 amplitudes in the same manner as described above for the P100. First, we computed an ANCOVA on N100 amplitudes, with stimulus (frequent and deviant), electrodes (C3, Cz, and C4), and modality (neutral, animal, and emotional) set as within-variables. Once again, inventory scores (BDI, STAI, and TAS-20) were covariates. Through this analysis, we only obtained a significant modality × stimulus × TAS [*F*_(2, 90)_ = 3.839, *p* = 0.027] interaction.

To clarify the significant modality × stimulus × TAS interaction, the participants were split into LOW and HIGH groups based on their TAS-20 scores. We then performed a 2 × 3 × 3 × 2 ANOVA on N100 amplitude values, with group (LOW and HIGH) as the between-subject factor, and modality (neutral, animal, and emotional), electrode (C3, Cz, and C4), and stimulus (frequent and deviant) set as within-subject variables. We observed significant effects for modality [*F*_(2, 96)_ = 8.807, *p* < 0.001] and stimulus [*F*_(1, 192)_ = 44.723, *p* < 0.001], as well as a significant modality x group interaction [*F*_(2, 96)_ = 5.082, *p* = 0.014].

As no significant result was obtained for the stimulus × modality × group [*F*_(2, 96)_ = 0.994, *p* = NS) and no electrode effects were found [modality × electrode × stim: *F*_(4, 192)_ = 0.605, *p* = NS; modality × electrode × stim × group: *F*_(4, 192)_ = 1.198, *p* = NS], we performed further analyses on the averaged amplitudes (mean of C3, Cz, and C4), as well as the averaged amplitudes with regard to the stimulus (mean of the frequent and deviant amplitude for AVE, AVA, and AVN). In order to better define these results, we subsequently performed a repeated measures ANOVA for each group (between factor), according to the modality (within factor). We obtained a modality effect [*F*_(2, 96)_ = 8.807, *p* = 0.001], a modality × group interaction [*F*_(2, 96)_ = 5.082, *p* = 0.014], and no group effect [*F*_(1, 48)_ = 0.027, *p* = NS]. *Post-hoc* Bonferroni tests showed that the highest amplitude was found in the AVE condition (−3.799 μV) and the lowest was observed in the AVN condition (−3.005 μV, *p* = 0.004). The AVA condition (−3.281 μV) differed from the AVN (*p* = 0.002) but not the AVE (*p* = 0.454) condition. Regarding the modality x group interaction, no differences were found for the LOW group [*F*_(1, 48)_ = 1.609, *p* = NS]. For the HIGH group, the highest amplitude was found in the AVE condition (−4.035 μV) and the lowest was observed in the AVN condition (−2.687 μV, *p* = 0.003). The AVA condition (−3.464 μV) differed both from the AVN (*p* = 0.0286) and AVE (*p* = 0.016) conditions (see Table 7).

**Table 7:** **Means and standard deviations (in parentheses) of N100 amplitudes for frequent and deviant stimuli together, in the “emotional” bimodal-AVE, “animal” bimodal-AVA and “neutral” bimodal task-AVN, for each group**.

	**Modality**	**Mean**
**LOW**		
	AVN	−3.324 (2.07)
	AVA	−3.098 (1.58)
	AVE	−3.563 (1.35)
**HIGH**		
	AVN	**−2.687 (1.64)**
	AVA	**−3.464 (1.33)**
	AVE	**−4.035 (1.79)**

*Significant amplitudes are indicated in bold*.

These findings suggest that more attentional resources were needed in the HIGH group to interpret “semantic” stimuli vs. neutral stimuli, which was more obvious when dealing with emotional stimuli. No differences were found in the LOW group regarding the modality of the condition, while in contrast, it appeared that more attentional resources were needed to decipher “semantic” stimuli in the HIGH group, which was even more obvious when dealing with emotional stimuli. Thus, it appeared that the N100 amplitude modulation was present, but only in the HIGH group. Similar analyses were conducted on N100 latencies but no significant results were obtained (*p* > 0.05).

#### P300

We performed ANCOVA on the P300 amplitudes, using stimulus (frequent and deviant), electrodes (P3, Pz, and P4), and modality (neutral, animal, and emotional) as within-variables. Inventory scores (BDI, STAI, and TAS-20) served as covariates. No significant results were obtained for either the amplitudes or latencies (*p* > 0.05; see Table 6 for values), which was consistent with the absence of behavioral effects. Indeed, P300 values are known to correlate with RTs, functionally referring to response-related stages (Polich, 2007).

#### Complementary analysis

***Rejected Trials***. We performed a 3 × 2 × 2 ANOVA on the rejected trials. Modality and stimulus were set as within-variables and the group served as the between factor. We observed no group interactions through this analysis: modality × group [*F*_(2, 96)_ = 0.993, *p* > 0.05] and modality × group × stimulus [*F*_(2, 96)_ = 2.537, *p* > 0.05]. These data indicate that the same number of “artifact” trials was rejected in each group.

***Correlations***. The TAS-20 is composed of 3 subscales: (1) difficulty identifying feelings, (2) difficulty describing feelings, and (3) externally-oriented thinking (Bagby et al., 1994a,b). We wanted to test whether some of these subscales might be related to specifically the P100 and/or N100 modulations that we observed in the AVE condition. To examine this possibility, we performed Pearson's correlations based on the 3 TAS-20 subscores and the P100/N100 amplitude means obtained from the AVE stimuli. Results are shown in Table 8.

**Table 8:** **Pearson correlations an *R*^2^ between the 3 sub-scores on the TAS-20**.

		**P100 AVE dev**	**N100 AVE dev**	**N100 AVE Freq**
**LOW**				
F1	Pearson correlation	0.115	0.226	0.209
	*p*	0.585	0.278	0.317
F2	Pearson correlation	−0.003	0.342	0.276
	*p*	0.989	0.094	0.182
F3	Pearson correlation	***−0.362***	0.269	0.259
	*p*	***0.075***	0.193	0.211
**HIGH**				
F1	Pearson correlation	−0.260	0.323	0.343
	*p*	0.209	0.115	0.093
F2	Pearson correlation	−0.065	−0.138	−0.039
	*p*	0.757	0.509	0.853
F3	Pearson correlation	**0.458 (*R*^2^ = 20.9%)**	−0.232	**−0.484 (*R*^2^ = 23.4%)**
	*p*	**0.021**	0.264	**0.014**

*F1, difficulty identifying feelings; F2, difficulty describing feelings; F3, externally-oriented thinking and the P100/N100 amplitude means in the AVE situation for frequent and deviant stimulations. Significant correlations are indicated in bold, tendencies in bold and italic*.

For the LOW group, a tendency for a negative correlation (*r* = −0.362, *p* = 0.075) was seen between the externally-oriented thinking subscale (F3) and P100 amplitudes for the AVE deviants (i.e., the higher the F3 score, the lower the P100 amplitude). For the HIGH scorers, F3 was significantly correlated (*r* = 0.458, *p* = 0.021) with the P100 amplitude for the AVE deviants (i.e., the higher the F3 score, the higher the P100 amplitude). The F3 was also negatively correlated (*r* = −0.484, *p* = 0.014) with the N100 amplitude for the AVE frequent stimuli (i.e., the higher the F3 score, the lower the N100 amplitude). Thus, it seems that the influence of the TAS-20 can be mostly explained by the F3 factor (externally-oriented thinking), at least with regard to the HIGH group for the P100 amplitude in the AVE deviants (*R*^2^ = 20.9%) and the N100 amplitudes in the AVE frequent stimuli (*R*^2^ = 23.4%).

## Discussion

In this study we investigated whether subclinical alexithymia could disclose particular emotional effects through the use of bimodal oddball tasks. In order to achieve this, we used a more sensitive paradigm than the classic unimodal oddball task, namely an audio–visual oddball task. The use of this tool already allowed us to index the subtlest subclinical differences (anxio-depressive tendencies) at the electrophysiological level, not revealed when unimodal stimuli were employed (Campanella et al., 2010, 2012). Through three different kinds of bimodal oddball tasks (emotional, animal, and neutral), we were able to explore the effect of high and low subclinical alexithymia on P100, N100, and P300 components.

Previous studies (Mann et al., 1995; Galderisi et al., 2008; Vermeulen et al., 2008) demonstrated that no behavioral differences could be detected when low- and high-alexithymic scorers were confronted with cognitive tasks, including emotional conditions. These findings suggest that at a subclinical level, an emotional processing deficit could remain behaviorally invisible, stressing the importance of using sensitive imaging tools, such as ERPs. Indeed, even in the absence of behavioral modifications, subtle alterations in ERPs have been shown to index minor cognitive restrictions (Rugg and Coles, 1995). In this study, examining the interaction between the TAS-20 values and the distinct bimodal tasks revealed specific ERP modulations. We found that high-alexithymic scorers presented greater amplitudes for early perceptive ERP components as compared with low-scorers when confronted with emotional oddball tasks (AVE). Moreover, our findings confirmed that no specific emotional effect was driven by depressive (BDI) and anxious (STAI) tendencies, as shown in Campanella et al. (2012).

We compared 3 kinds of bimodal stimuli [emotional (AVE), animal (AVA), and neutral (AVN)]. We observed that high-scorers displayed an enhanced visual P100 component in response to deviant stimuli in the AVE condition, which did not occur in the AVN and AVA conditions. However, no difference between the AVE and AVA conditions emerged from the low-scorers. High-scorers also showed enhanced N100 amplitudes for both frequent and deviant stimuli presented in the AVE condition, whereas again no difference was observed when analyzing low-scorers. Furthermore, independently of the group, AVN was the condition that generated the lowest amplitudes for P100 and N100, suggesting that semantic conditions (AVA and AVE) require more attentional resources to be processed. Additionally, in both groups emotional stimuli systematically exhibited higher amplitudes. This result can be interpreted by the emotional salience of the stimuli in terms of survival, reproduction, and procreation, compared to other kinds of stimulations (Schupp et al., 2007).

Overall, the alexithymia-high group demonstrated modified processing of the emotional condition, which translated into higher amplitudes in the early visual treatment and extended to the early auditory processing of the stimuli. These results are in line with findings by Wehmer et al. (1995) and Franz et al. (2004) about unimodal emotional situations and supports the idea that, contrary to behavioral data suggesting an incapacity to detect and process emotions, ERPs data suggest that these subjects are not blind, at a perceptive level, to emotional content, but may request some kind of “hyperactivation” (physiological or electrophysiological) in order to process this information and correctly perform. Thus, they are able to experiment emotions, but at the cost of a modified central processing of it, as exhibited by the ERPs modulations. To compensate for their emotional disturbances, subclinical alexithymic subjects actually allocate abnormal attentional resources to successfully perform tasks. For this reason, no behavioral differences (reaction times and correct response rate) could be identified. Indeed, several studies have indicated that emotional processing is not entirely automatic but competes with attentional demands. Thus, emotional stimuli reciprocally influence attentional processes (Pessoa et al., 2002, 2005; Bradley et al., 2003; Sabatinelli et al., 2005). Therefore, alexithymic individuals can perceive emotional stimuli and are not “blind” to emotional information; however, they require a deeper, more intense cognitive process for processing the stimuli. This could be due to doubts in their minds with regard to the meaning of the emotion presented, or perhaps they are less interested in emotions (less familiar with them) (Grynberg et al., 2012).

Interestingly, P100 modulations were only present for deviant stimulations, with some differences occurring between the high- and low-scoring groups. For the alexithymia-low group, the only distinction that could be made was between the neutral condition and the emotional one, suggesting that the “semantic” content of the stimuli required deeper processing (regardless of whether the stimuli was emotional or animal). Indeed, high-complexity pictures (compared to low) are known to require additional attentional resources for processing details (Bradley et al., 2007). However, for the high group, an “emotional effect” occurred (i.e., the emotional condition was harder to deal with because more attentional resources were necessary to interpret or decipher the emotional content). This effect could be indexed by larger bimodal P100 amplitudes in response to emotional conditions, compared to the animal and neutral stimuli, as has been previously described in the literature for unimodal visual situations (Pollatos and Gramann, 2011; Singhal et al., 2012). Therefore, our data suggest that a very early attention modulation occurs in response to emotional stimuli, as high-alexithymic-scorers displayed larger amplitudes to pictures that were more “complex to process.” Likewise, processing was easier for stimuli that were lower in affective relevance (AVA and AVN) and did not require selective processing (Pastor et al., 2008).

This deficit extended to the auditory domain, but only for the alexithymia-high group (for both frequent and deviant stimulations). Indeed, the N100 amplitudes were gradually larger from the AVN to AVE conditions, suggesting a specific “emotional effect.” These enhanced N100 amplitudes appear to confirm the idea that deficits in emotional prosody exist in alexithymic subjects, at a behavioral level (Schäfer et al., 2007). For instance, when Goerlich et al. (2011) used emotional prosody (music and words with emotional connotations), they did not observe any behavioral differences; however, using affective categorization of happy and sad prosody and music targets they could elicit a negative correlation between alexithymia and the N400 amplitude. Another recent study of Goerlich et al. (2012) also demonstrated larger N100 amplitudes in high TAS-20 scorers in response to deviant emotional prosodies, whereas no behavioral differences were found between the low- and high-alexithymic groups. They also found that alexithymia is related to generally blunted neural responses to speech prosody (Goerlich-Dobre et al., 2013). Our results are consistent with the theory that subclinical alexithymic subjects exhibit attenuated basic emotional processing, involving a reduced early detection of emotional salience that requires more attention for detection of changes in emotional acoustic cues.

Another interesting finding of this study was that only deviant stimuli led to changes in the visual P100 component, whereas both frequent and deviant cues led to N100 modulations. A possible interpretation of this result is that, as mentioned by Joassin et al. (2004), the quantity of available information at stimulus onset is not equal for faces and voices. It has been proposed that auditory information unfolds over time for voices and that this time-based asynchrony can lead to an interference effect (Calvert et al., 2001). This might be why high-scorers require more resources to process general auditory stimulations, particularly for the more complex “emotional” condition (AVE). Further studies should be designed to investigate this topic in-depth.

At later stages of the information processing stream, no differences in P300 modulations were observable between low- and high-alexithymic individuals. Since P300 functionally corresponds to response-related stages (Polich, 2007), these data are in perfect agreement with the absence of behavioral modifications. This suggests that subclinical alexithymic subjects are able to compensate for their emotional deficits in later decisional levels, as they performed the task equally to low-scorers. However, this “normal” performance by alexithymic individuals requires more visual and auditory attentional resources devoted at early stages of the information processing stream. Further studies analyzing alexithymic subjects with clinical TAS-20 values (>61) should be performed in order to verify whether early deficits would extend to later ERP components in these more extreme cases. Indeed, it would be interesting to apply our bimodal emotional task in clinical populations, as the early emotional alterations (P100 and N100) could constitute a marker of predisposition for the development of later clinical alexithymia. Thus, these data may have an important clinical relevance. If the behavioral deficits presented by clinical alexithymic individuals have an attentional origin, then cognitive remediation procedures targeting the deeper attentional processing of emotions could be envisioned.

Despite the potential clinical relevance of the data, the limitations of our study must be outlined. First, our sample size was modest (50 participants). Second, the TAS-20 scale is an auto-evaluative questionnaire, which could be paradoxical for individuals suffering from difficulties in identifying and describing their own emotions. To address this important issue, future studies could employ observer evaluations, such as those conducted in the Toronto Structured Interview for Alexithymia (TSIA, Bagby et al., 2006) or the Observer Alexithymia Scale (OAS, Haviland et al., 2000). Third, we made the subjects fill out the questionnaires (BDI, STAI, and TAS-20 assessing emotions) before the ERP session, so it is possible that their attention toward emotional processing could have influenced the way that they processed the emotional condition. Fourth, we only used negative (sad) and neutral stimuli in this study; however, alexithymia has been described to relate to a large variety of basic emotions (e.g., sadness, happiness, fear, anger, disgust, and surprise) (Jessimer and Markham, 1997; Prkachin et al., 2009), as well as neutral stimuli (Lane et al., 2000). Thus, it would be interesting to link these particular difficulties with specific ERP modulations. Finally, it is possible that alexithymia could negatively impact performance in the fast processing of emotional information, as described in our paradigm. With regard to this, a study by Parker et al. (2005) used a signal–detection design that allowed participants to judge neutral or negative facial expressions under slow and quick presentation conditions. Indeed, the alexithymia component of difficulty in describing feelings was inversely correlated with the capacity to detect negative emotional expressions in the rapid condition.

Our findings support the utility of a more sophisticated oddball (i.e., an audio–visual design) for revealing subtle subclinical differences in healthy populations. Our results are in line with previous reports that have suggested atypical attentional resource allocation in alexithymic individuals during negative and neutral bimodal stimuli. Notably, this allocation would allow these subjects to compensate a perceptive deficit (auditory and visual) and successfully perform tasks. Therefore, we highly recommend the use of bimodal stimulations for future studies, as they allow the extraction of more accurate knowledge about the cognitive processing of emotions (Maurage and Campanella, 2013). This is necessary to create and develop an adequate rehabilitation plan, as alexithymic patients unsuccessfully respond to psychological treatments that center on introspection, emotional consciousness, and/or close alliances with therapists (Lumley et al., 2007). Moreover, they rarely engage in treatment recommendations (Ogrodniczuk et al., 2011). As a matter of fact, our correlational results revealed that the P100 and N100 modulations were mostly associated with the operational thinking subscale of the TAS-20 (e.g., prefer talking about daily activities rather than feelings). As mentioned before, recent ERPs data, including the present study, reveal that alexithymics, in any case subclinical subjects, are finally not blind to emotion, and are able to detect and process it, so the whole concept of alexithymia is challenged. As suggested by Franz et al. (2004), perhaps alexithymics avoid the processing or the expression of affective states. Nevertheless, the externally-oriented thinking style seems to remain a stable feature of the disorder, and therapeutic interventions could successfully target this characteristic. Knowing that alexithymics are more likely to seek healthcare (Joukamaa et al., 1996), this information could be useful for engaging alexithymic individuals in externally-focused interventions that might guarantee greater adherence to structured exercises and behavioral counseling. From this perspective, alexithymia could be redefined as a deficit in emotional aptitudes that could be learned or trained, among other things, through treatment (Lumley et al., 2007). For example, Levant (2001) developed a cognitive–behavioral method in which alexithymics were taught to learn emotional vocabulary, label affective situations, observe their own symptoms, and connect emotional labels to their symptoms. It is important that future longitudinal studies investigate the long-term effects of such cognitive–behavioral techniques in alexithymia treatment. In this regard, it would be interesting to evaluate whether early ERP amplitudes to emotional stimuli decreased over time during these follow-up studies.

### Conflict of interest statement

The last author is funded by the Belgian Fund for Scientific Research (F.N.R.S., Belgium), but this fund did not exert any editorial direction or censorship on any part of this article. The other authors declare that the research was conducted in the absence of any commercial or financial relationships that could be construed as a potential conflict of interest.
